# Can tourists become disciples? The formation and mechanism of place conversion in traditional Chinese villages

**DOI:** 10.3389/fpsyg.2023.1301127

**Published:** 2023-12-18

**Authors:** Luyao Xu, Lei Shi, Xin Wang, Yuliang Wan, Zhuo Fan

**Affiliations:** ^1^School of Geography and Tourism, Chongqing Normal University, Chongqing, China; ^2^School of Business, Wuxi Taihu University, Wuxi, China

**Keywords:** rootedness theory, place conversion, formation mechanism, traditional village, genius loci, place attachment

## Abstract

Place attachment has been extensively studied in the field of tourism. However, it is important to recognize that place attachment alone may not fully capture the emotional expression of tourists’ spiritual beliefs and their sense of belonging to a destination. Therefore, a more comprehensive attachment theory is needed to encompass higher dimensions. This article focuses on the ancient Chinese villages of Yantou, Cangpo, and Furong from a tourist perspective, and introduces genius loci in architectural phenomenology. We set out to expand the study of spiritual dimensions of place attachment on the basis of local attachment, redefining tourists’ place connection and putting forward the new concept of place conversion. A qualitative analysis of online text using Rooting theory was conducted to condense 22 categories into 6 main categories and establish a model. The study shows that the mechanism of place conversion is composed of situational perception (place perception and place identity), physical and mental immersion (spatio-temporal immersion and place conversion), and behavioral willingness (tourists intention and conversion behavior). The study has implications for the conservation and development of traditional villages and future research on the spiritual experiences of tourists.

## 1 Introduction

In the third year of the Yongchu era of the Jin Dynasty (422 AD), Xie Lingyun was exiled to Yongjia County, known at that time as the “southern barbarian land.” Xie Lingyun had been born into a prominent and distinguished family of the Eastern Jin Dynasty. A talented individual and the first landscape poet in China, he was also a renowned literary figure, Buddhist scholar, and traveler. He wrote many timeless landscape poems about Yongjia and became the pioneer of Chinese landscape poetry. For example, the phrase “拂衣遵沙垣，缓步入蓬屋。近涧涓密石，远山映疏木” translates as:

I lightly brush my clothes as I walk slowly along the sand wall and enter the thatched house. Nearby, the stream in the ravine flows gently over the dense rocks, and in the distance, the mountains reflect the sparse trees.

The Nanxi River extends for three hundred miles of poetic scenery, together with ancient villages in Tang Dynasty style with Song Dynasty charm. When Xie Lingyun explored the ancient villages – concentrated settlements where people live together – along the Nanxi River, he likely experienced a sense of contentment and abandonment, similar to the people in the legendary Peach Blossom Spring of Wuling. The tranquility, simplicity, freshness, and warmth of these villages comforted him during his time of loneliness and confusion, healing the wounds in his heart. Today, these traditional villages retain the pure and secluded atmosphere of an idyllic paradise, attracting tourists and soothing their souls.

The human-place connection is a complex phenomenon with many meanings. Three of the meanings are well recognized: the physical/objective environment; people’s experiences/behaviors, and socially constructed meanings of places (Stedman, 2003b). The core concept of place attachment dominates the research field of people and place (Tuan, 1974, 1977). Tourists may develop an emotional connection to a village during their visit, which scholars mainly explain in terms of sense of place and place attachment (Kastenholz et al., 2020). Some scholars consider that rural tourism is a way of meeting the unformalized and non-ritualized spiritual needs of modern tourists, and that these spiritual experiences require further investigation (Sharpley and Jepson, 2011; Jepson and Sharpley, 2015). Thus, this study aimed to explore the spiritual and mental experiences that villages bring to tourists, and to develop a new conceptualization beyond place attachment.

Contemporary tourism divides the study of spirituality into two perspectives: (1) religious tourism and tourist pilgrimages, where participants are motivated by religious reasons, and (2) tourists as secular pilgrims who use tourism as a source of spiritual purification (Digance, 2006; Kim et al., 2020: p.25). Villages have landscapes, heritage, and traditions that create spaces that are perceived as idyllic in contrast to cities (Bell, 2006; Kastenholz and Lima, 2012; Kastenholz et al., 2012; Carneiro et al., 2015). First, nature is viewed as sacred and tourists may describe “an encounter with the soul of nature” and emotions such as “wonder, awe, wholeness, harmony, ecstasy, transcendence and solitude” (Timothy, 2012: p.38). Second, villages are constructed as a paradise for people’s spirits and souls, and a place of nostalgia where tourists can find their own spiritual home and way of life in the countryside. The pressures of life and work in modern society burden people, making rural tourism an important way for individuals to escape from the chaos of everyday life and find inner peace. The significance of nostalgia evoked by villages lies in the transformation of history into individual and collective myths, and the reliving of lost childhood days (Boym, 2007; Chen et al., 2019; Chylińska, 2022). Finally, villages form the basis of human survival, and tourist behavior manifests in loyalty and emotional solidarity when tourists escape from the city and return for a sojourn in the countryside (Oppermann, 2000; Wang and Chiou, 2019; Cheng et al., 2020).

This study introduces the concept of genius loci and explores the interaction between tourists and the spirit of the village, and the spiritual experiences of tourists. The concept of genius loci originated in ancient Rome, where it was believed that the soul of each person could give life to both people and places, determining the characteristics and essence of a place (Norberg-Schulz, 1979: p.18). Farming in rural areas ensures necessary produce and life for local residents. Thus, these places carry unique memories. Over thousands of years, the villages have been endowed with meaning and connotation by local residents, gradually acquiring specific characteristics and essence. Thus, the reasons for selecting the concept of genius loci in this study are as follows. First, the spirit of rural places conveys a strong sense of place to tourists, providing them with a spiritual experience that meets their spiritual needs (Christou et al., 2019). Second, research on the spirit of village place is effective in explaining tourists’ sense of identity, direction, and belonging, providing theoretical support for the study of tourists’ spiritual experiences.

Tourists describe their strong emotional attachments to villages as characterized by a sense of spiritual belief and belonging. Their behaviors also demonstrate a deeper level of involvement and proactiveness, such as revisiting, sharing, recommending, and even residing in villages. Ultimately, tourists visit traditional villages for the tourism experience, and for social and psychological reasons, such as gaining a sense of identity, belonging, and direction. Therefore, this paper argues that it is difficult to capture the essence of place attachment by defining it solely in terms of emotion. Drawing on the terminology of religious studies, we define tourists’ emotional expression, beliefs, and behaviors related to place as “place conversion.” Conversion is a Buddhist term meaning that the body and mind are directed, attached, and empowered by the Three Jewels (Buddha, Dharma, and Sangha). Thus, place conversion is the belonging and attachment of tourists’ spirit, faith, and mind in the village space – the ultimate expression of genius loci and an interpretation of the tourist experience.

In China, rural settlements are also known as traditional villages, often reflecting the traditional style, local characteristics, and folk customs of specific historical periods. These villages have great historical, cultural, and architectural value (Guo and Sun, 2016). In this study, we used the ancient villages of Yantou, Cangpo, and Furong in the Nanxi River Basin as examples to explore the spiritual world of tourists in ancient villages, and further investigate the essence of travel experiences. This study aimed to provide a theoretical basis for understanding tourists’ spiritual belonging, and to ensure the protection and development of traditional villages.

## 2 Theoretical background

### 2.1 Place attachment

The emotional connection of human-place interactions – place attachment – is an important topic of research in human geography, environmental psychology, and sociology. Theoretical research in this area is mainly concerned with the concept, dimensions, and influencing factors of place attachment. Most studies of place attachment use quantitative research methods, with few qualitative studies available. Place attachment is defined as the degree to which an individual evaluates or identifies with a particular environmental place. The theoretical framework of place attachment consists of two dimensions: place identity and place dependence (Williams and Roggenbuck, 1989; Williams et al., 1992). Place identity refers to “the strong emotional attachment of an individual to a particular place or environment.” The tourism environment allows individuals to express and confirm this identity, increasing one’s sense of belonging to a destination (Tuan, 1980; Kyle et al., 2004). Place dependence (or functional attachment) is defined as “how well an environment serves goal attainment given the range of alternatives available,” and places that meet multiple needs often lead to deeper place dependence (Stokols, 1981; Jorgensen and Stedman, 2001). Some scholars have integrated the concept of place attachment into a three-dimensional “person-process-place” organizational framework where the person dimension refers to the meaning of individual and collective decisions; the psychological dimension includes emotional, cognitive, and behavioral components; and the place dimension emphasizes the characteristics of place (Scannell and Gifford, 2010). The environmental ensemble, people-in-place, and common presence affect people’s place attachment (Seamon, 2013), in turn affecting tourist satisfaction and loyalty (Rollero and De Piccoli, 2010; Vada et al., 2019).

Some places have a great impact on tourists’ inner spirituality – as in the case of religious pilgrimages (Lois-González and Santos, 2015). Places that have strong historical significance or that trigger significant historical transitions also strengthen tourists’ place attachment, e.g., visitors to the Forbidden City deepen their place attachment when they perceive its authenticity (Wu et al., 2019). At present, place attachment is difficult to explain, and the phenomenon of soul impact on the human mind through place is hard to express. Thus, there is a need to further explore theories of place attachment to explain these phenomena.

### 2.2 Genius loci

The Norwegian architect Christian Norberg-Schulz proposed the concept of genius loci, which has been widely used in the field of architecture and in the creation of space, becoming key to the creation of tourist spaces (Loukaki, 1997; Markevičienë, 2012). There is a strong interaction between space and human emotions. On the one hand, space can transmit genius loci belonging to the place, and on the other hand, genius loci transmitted through space generates responsive emotions. Genius loci is often combined with the culture, history, religion, and clan of heritage sites because the original source of genius loci is inclined to faith. Genius loci can be expressed in places with a strong historical and cultural atmosphere, such as the World Heritage town of Bhaktapur, Nepal, which is described as having a sense of sacredness, community, history, and tranquility (Silva, 2015). These unique place “spirits” become the identity of the place and result in unique experiential qualities for tourists. Atmosphere, sense of place, and genius loci in religious spaces can have an emotional and psychological impact on visitors, enhancing tourists experiences and the emotions felt in such places (Monzavi and Günçe, 2019).

At the same time, researchers have argued that the spirit of place changes with place and time, and therefore, certain places need to be preserved (Christou et al., 2019; Jiang and Lin, 2022). For example, the spirit of place is inherited and developed over time, and the Buddhist-themed spirit of place in the World Heritage city of Anuradhapura, Sri Lanka, thrives in the architecture, landscape, villages, and cities, illustrating spiritual sustainability (De Silva, 2023). Traditional villages are special tourist destinations associated with a unique local culture and connotation. Exploring the spirit of place in traditional villages can, to some extent, preserve and develop rural cultural history.

### 2.3 Cognition-affect-conation theory

The theoretical framework of this study draws on the cognition-affect-conation (CAC) model of personal cognition (Hilgard, 1980). This model describes a progressive process in which cognition influences emotion and, thus, behavioral intentions, and is widely used in marketing. Attitudes refer to affective-emotional response activities; cognition refers to beliefs, knowledge, and thoughts related to attitudinal goals; and intention refers to behavioral intentions and behavioral loyalty. From a psychological perspective, human perceptions and attitudes toward place have three basic dimensions: affective, cognitive, and intentional, and research on sense of place focuses on these areas. In sense of place research, place identity in place attachment is considered as the cognitive dimension; emotional attachment is considered the affective dimension; and place dependence or loyalty is considered as the intentional dimension (Park, 1996; Pritchard et al., 1999; Kyle et al., 2006). This theory often relies on structural equation modeling to test the validity of the path by constructing a cognitive-emotional-intentional framework with corresponding variables. In tourism destination research, there is an influential relationship between tourists’ cognition, emotion, and destination brands based on loyalty (Tran et al., 2023). CAC theory elucidates the interaction mechanisms between destination attachment formation and tourist psychology, revealing 3 psychological pathways (Qu et al., 2021). Therefore, this theory can be used in the study of psychological affect and formation mechanisms. The present study uses this framework to explore tourist place conversion formations of action, and to study affect – and, thus, tourist behavioral intentions – by analyzing tourists’ perceptions of the countryside.

## 3 Research design

### 3.1 Study region

**FIGURE 1:**
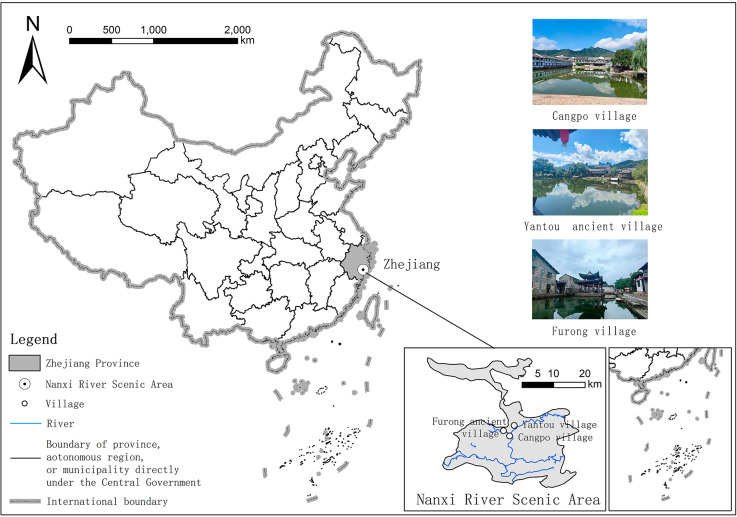
Overview map of study region. Village photos taken by the authors.

The Nanxi River Ancient Village refers to a village in Yongjia County, Wenzhou City, Zhejiang Province, China (Figure 1). It was built during the Southern Song Dynasty and consists of residential houses, ancestral halls, academies, village gates, temples, pavilions and archways with artistic, cultural, and historical significance. This region was chosen for the following reasons:

(1) The Nanxi River Ancient Village has a rich cultural heritage and is widely recognized as a traditional Chinese settlement. It has a unique agricultural and educational culture – it developed the important Yongjia School during the Song Dynasty, a historical library of Chinese rural culture. (2) The village architecture has a unique and distinctive style, and the village is carefully located and meticulously planned, making it one of the four major types of Chinese residential areas. The environment is rustic and harmoniously integrated with nature. Further, the village carries the historical and cultural memory of the Chinese nation and evokes a sense of nostalgia for tourists, making it conducive to the study of local attachment and the spiritual significance of village places.

We selected three representative villages in the Nanxi River Basin, all of which are recognized as Chinese landscape villages. Furong Ancient Village is the most famous and representative village in the region, and it embodies the architectural concept of “Seven Stars and Eight Dipper.” The village is surrounded by cobblestone walls, resembling a small castle. Cangpo Village exudes a strong agricultural and educational culture. It was planned according to the principles of Yin and Yang and the Five Elements, with the “Four Treasures of the Study” as the guiding layout. Yantou Village is located on the west bank of the middle reaches of the Nanxi River, between Cangpo and Furong. The “Agricultural and Educational Corridor” in the village is a pathway paved with goose eggs, allowing tourists to experience the profound history and culture of ancient civilizations while walking on Lishui Street.

### 3.2 Research methodology

Grounded theory, first proposed by Glaser and Strauss in 1967, refers to the process of extracting theories from raw data through data collection, analysis, and coding, followed by innovation and refinement of the theories (Glaser and Strauss, 2017; Bakker, 2019). It is a research strategy that allows for in-depth exploration of phenomenological and sociological theories, and has been widely applied in the fields of management and tourism studies. The approach requires a shift in focus from the objects of tourism to the researchers themselves, emphasizing diverse perspectives. This application of grounded theory highlights the scientific and explanatory value of the approach in understanding tourists’ subjectivity, and therefore, grounded theory was judged to provide a solid foundation for this study.

The coding process in grounded theory includes open coding, axial coding, and selective coding, which, respectively, involve conceptualization, categorization, and theorization. Grounded theory has advantages in analyzing research areas that lack – or have insufficient – theoretical explanations. Previous studies have used grounded theory to analyze the use of historical mosque spaces, illustrating the mechanisms of interaction between soundscape elements, spatial functions, and local identities (Yilmazer and Acun, 2018). The purpose of this paper was to explore the place conversion mechanism of tourists in the rural space of traditional villages. This mechanism lacks theoretical support, and the bottom-up analytical approach of grounded theory was, thus, well suited for this exploratory study. We followed the research procedures of grounded theory and used the qualitative software NVivo12 for analysis.

### 3.3 Data sources and processing

This study used online texts from travelers to the Nanxi River Ancient Village as the data source. Well-known domestic travel websites, Ctrip and Mafengwo, were selected for the study. The selection criteria for the texts were as follows: (1) The travelogues should include at least one case study from either Yantou, Cangpo, or Furong Ancient Village. (2) The comments should be detailed, not copied and not limited only to picture descriptions. (3) The texts should be complete, including the author’s thoughts, feelings, and psychological activities, and have a high click-through and reply rate. Using the keywords “Nanxi River Ancient Village,” “Yantou Village,” “Furong Ancient Village,” and “Cangpo Village,” searches were conducted on Mafengwo and Ctrip. The above criteria were applied to the selection process, and travelogues containing content from other villages in the region were excluded. A total of 43 travelogues were obtained from the two websites: 12 from Ctrip (numbered A01−A12) and 31 from Mafengwo (labeled B01−B31). In addition, there were 312 comments on the Ctrip travelogues from Yantou, Cangpo, and Furong Ancient Village, labeled C01−C312. Out of these, 40 travelogues and comments were used for encoding, while A12, B30, and B31 (3 in total) were used for saturation testing.

## 4 Rooting theory analysis

### 4.1 Open coding

Open coding is the process of reading through text materials sentence by sentence, identifying key items, events, and themes, annotating preliminary concepts, and then exploring categories based on those preliminary concepts. In this study, a total of 945 reference points were coded, and after multiple rounds of coding and comparison, 115 concepts were identified. Based on the inherent connections between the concepts, related concepts were grouped together and named accordingly. A total of 22 categories were identified. Selected examples of the coding are shown in Table 1.

**TABLE 1:** Open coding and its categories.

Num	Original text	Conceptualization	Categorization
B18	Historical records indicate that there were two major waves of migration to Nanxi River in the past: one during the Jin Dynasty and another during the period of late Tang Dynasty and Five Dynasties and Ten Kingdoms.	Long history of village establishment	Historical significance
C239	The Huifeng Pavilion was built for Xia Huiying, the fourth-generation Chinese painter, who had a special affection for the landscapes of Nanxi River. The painter created numerous works depicting the beauty of Nanxi, forming a bond with the ancient village.	Commemoration of great men	
A4	On the south side of Dongchi Village’s wall, there are two ancient cypresses planted by Li Xizhai, the lord of Cangpo Village during the Song Dynasty more than 800 years ago. They are still thriving today. The “Ancient Cypress” on the monument next to the parking lot at the entrance of the village refers to these two trees.	Elemental history	
A11	The people in the ancient village seem to have stayed in the past time, in today’s fast-paced world. Coming here for a walk, my heart becomes calm, and my soul finds solace.	Heart contemplation	Conversion of the heart
B29	When I first saw the traditional village of Nanxi River, I was mesmerized by its beauty, and I could not help but want to know more about it, even more.	Heart awakening	
C191	Walking slowly along the small path paved with cobblestones, a gentle breeze brushes my cheeks, healing my soul. Softness and relaxation fill the air.	Heart healing	

### 4.2 Axial coding

The central idea of main axis coding is to organize new perspectives and themes by creating an axis based on the concepts and categories identified through open coding. This involves starting from the categories developed through open coding and using causal, temporal, and semantic relationships as connections. We extracted 6 main categories from the initial 22 concepts. These categories included place perception, place identity, spatio-temporal immersion, place conversion, tourist intention, and conversion behavior. Table 2 summarizes the categories and provides further details.

**TABLE 2:** Results of axial coding.

Main category	Corresponding categories
Place perception	Lifestyle, agricultural, social and cultural ambiance
Place identity	Architectural art; cultural, historical and constructed significance
Spatio-temporal immersion	Time travel, immersive atmosphere, indulging in beautiful scenery, time, enjoyment
Place conversion	Spiritual conversion, conversion of the heart, faith conversion, physical conversion
Tourist intention	Recommendation intention, revisit intention, share intention
Conversion behavior	Lingering and unforgettable experiences, temporary stay and exploration, settling back in the hometown

### 4.3 Selective coding

The storyline is a description of the phenomenon under study based on the collected data, the completed concepts, categories, and relationships. By comparing, integrating, and refining the logical relationships between the 6 main categories and verifying their relationships, we discovered the core concepts that can explain the concepts, categories, and main categories. This study constructed a mechanism of place conversion by connecting the stories in a series that included the 3 dimensions of situational perception, physical and mental conversion, and behavioral willingness.

**FIGURE 2:**
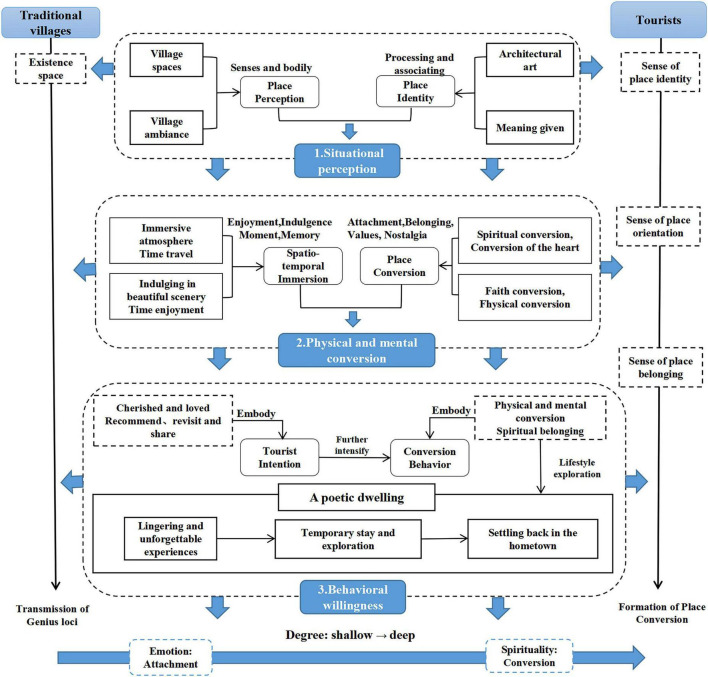
Mechanism of place conversion.

Figure 2 shows how this study searches for spiritual and psychological connections between tourists and traditional villages. Traditional villages convey genius loci as an existential space, and tourists generate spiritual and psychological experiences through situational perception, physical and mental immersion, and ultimately express them through behavioral willingness. This process is embodied in tourists’ spiritual sense of identity, sense of orientation and sense of belonging. Therefore, three core concepts need to be explained first: 1. Situational perception: tourists in the process of individual perception of the ambiance of traditional villages and traditional village places to produce a sense of identity; 2. Physical and mental immersion: tourists immersed in traditional villages and produce spiritual beliefs and soul belonging to the expression of emotions; 3. Behavioral willingness: tourists are very fond of villages, and thus find a suitable way of life, through different ways of living and belonging to the village, and finally through behavioral willingness. They find their own way of living and belong to the village space through different ways of living. Secondly, this study started from the tourists’ spiritual “orientation” and “identification,” and explored the reasons and processes for the formation of place conversion from the tourists’ sense of place identity, place orientation, and place belonging. Sense of place identity refers to people being in the atmosphere and space of the village, perceiving the village place through their 5 senses and body, gazing at the village buildings, giving the village space meaning, and generating a sense of identity for the village place through the processing of associations. The sense of place orientation refers to people being in the place, understanding where they are and the spiritual function of passing through earth, exploring the value of life, and becoming a part of nature. The true sense of belonging to the place is the complete development of these two spiritual functions, seeking nostalgia in the village, living a life of longing for seclusion, lamenting life, achieving the state of unity between heaven and man, getting spiritual satisfaction, seeking attachment and belonging to the spirit and mind, and realizing physical and mental conversion. In the formation process of tourists place conversion, with the gradual sublimation of the emotions of tourists, tourists loyalty to further deepen, people seeking poetic habitat conversion behavior from shallow to deep for the stays, living, and residing permanently, corresponding to the lingering, temporary stay and setting back in the hometown.

The theoretical saturation test was used to determine when to stop sampling. The constructed model was tested for saturation using the remaining 3 travelogue texts. No new categories or relationships were generated, indicating that the model of the role of places of refuge had been extensively developed, and that the model constructed in this study achieved theoretical saturation. For example, the following excerpt aligns with the concept of “spatio-temporal immersion” in Table 2.

Quietly, the ancient Qingshan Academy tells its long history to every tourist, and in that moment, I could almost hear the melodious sound of students reading. The ancient waterwheel flows silently for thousands of years (A12).

Similarly, “Far different from Lijiang, Fenghuang Ancient City of vulgarity, the village seems to be the beauty of life, subtly hidden. The breath of life can inadvertently be found!” (B31) aligns with the concept of “lifestyle ambiance → place perception.”

## 5 Mechanism of action model

### 5.1 Situational perception

#### 5.1.1 Place perception

Traditional villages have a unique spatial structure. In architecture, the concept of existential space includes both space and characteristics. Norberg-Schulz (1979: p.7) states that place means “a totality of concrete objects that have a material essence, shape, texture and color. The totality of these objects determines a “character of the environment,” the essence of place.” A place is a special type of space with its own characteristics, often referred to as atmosphere. Rural areas are spaces with atmosphere, and traditional village spaces are able to convey this atmosphere to tourists, creating a sense of place for them. Our study suggests that tourists’ sense of place is developed through their perception of the rural spaces of the Nanxi River village, including its agricultural and cultural aspects, and the local atmosphere of the village life and community.

Traditional villages provide produce and meet the vital needs of daily life for local residents. When tourists enter the village, they experience a rural agricultural landscape that is completely different from the city. The pastoral atmosphere conveys a strong agricultural ambiance to tourists. Visitors observe the slow pace of life and sense the peace and quiet of the village. Tourists envy the leisurely and cozy atmosphere of the village lifestyle.

The elderly woman washing clothes by the pond in front of the old house, and the old man sitting on a stone bench in front of the door, smoking a cigarette and taking a break. Being in this environment, I feel everything here is so peaceful, harmonious, and tranquil (A13).

Going to the rice fields outside the village to see the golden wheat ears. The heavy wheat ears are filled with the joy of a bountiful harvest. As the wind blows, the waves of wheat roll, creating a rural landscape painting of sowing in spring and reaping in autumn (A7).

Chinese culture has a long history, originating in the vast countryside. Traditional villages are places where local residents live and work, and also the source of their culture. Over the course of thousands of years, the Nanxi River Ancient Village has developed a unique culture of farming and studying, folk customs, landscape, and local culture. Culture is intangible, but it can be felt through tangible carriers when experiencing the local cultural atmosphere. In traditional villages, the presence of leisure and social spaces brings people closer. Tourists can see local residents gathering and chatting under ancient trees, in waterside pavilions and village squares, and on stages, creating a unique rural scene. The simple and down-to-earth local residents lend a rustic atmosphere to the village.

Here you can quietly read about the culture of farming and reading, and feel the atmosphere of the countryside, which is decidedly different from the city (A10).

No matter where you go, there are people who are willing to communicate with you proactively, inviting you into their homes and offering you the snacks they made. It is truly touching (C228).

#### 5.1.2 Place identity

The concept of identity originated in psychology and has gradually expanded into the field of sociology. Proshansky argued that place identity refers to certain aspects of the self that develop within the physical environment. Place identity is a part of self-identity (Proshansky, 1978). Places are unique, and when a space is imbued with specific cultural, historical, and spiritual significance, it emits unique spiritual qualities. The spirit of a place becomes the life force that evokes cultural memory and historical experiences, awakening a sense of belonging to the place and the past. Therefore, people develop a strong sense of identification with villages that possess the spiritual qualities of a place.

Schultz (1979: p21) associates the characteristics of a place’s spirit with a sense of direction, identification, and belonging. Places are often associated with the cultural memory of individuals and communities. Natural locations acquire historical and cultural significance through human activities, becoming places. People define their home within their environment, establishing a connection between themselves and their surroundings. Therefore, architecture serves as a tangible expression of village art and embodies the cultural and historical essence of a village. Village buildings are quaint and exquisite, often constructed using wood and cobblestones, with intricate carvings, and tourists enjoy the artistic charm of the architecture. Additionally, the planning and construction of ancient residences in the Nanxi River region respects the spirit of nature and culture, creating an ideal living environment with complete harmony between humans, nature, and the divine.

The architecture of Cangpo Ancient Village is a condensed representation of ancient Chinese culture, with its overall layout designed according to the Five Elements, Eight Trigrams, and the Four Treasures of the Study (A10).

Furong Village is the oldest of the villages in Nanxi River, planned and designed based on the concept of the Seven Stars and Eight Dipper, symbolizing the correspondence between the stars in the sky and the people on the earth (B17).

Architecture is a symbol of individual memory for humans and a symbol of collective memory for human societies. Through the spiritual essence conveyed by architectural art, tourists awaken collective memory and historical experiences, together with their sense of identity, community, and nationality. Therefore, villages hold a significant spiritual meaning. Under the influence of tourists and society, villages are endowed with the spiritual meanings of an idyllic paradise and a haven of memories. On one hand, villages become a utopia for tourists to escape the activity of the city and seek spiritual belonging. On the other hand, they are a place for tourists to carry personal and collective memories and seek a sense of spiritual belonging.

Everyone works from sunrise to sunset, living a simple rural life, as if the outside hustle and bustle has no influence here. It truly feels like a utopia, allowing people to return to nature and enjoy life (C177).

The ancient village by the Nanxi River is simple and natural, touching the traces of history and reminiscing about childhood memories, sending nostalgia and emotions to this place (A8).

### 5.2 Physical and mental conversion

#### 5.2.1 Spatio-temporal immersion

Flow theory, first proposed by the American psychologist Csikszentmihalyi, explains how people filter out irrelevant perceptions to achieve a state of immersion. The core concept of this theory is the flow experience (Parsa and Cobanoglu, 2011; Rose et al., 2012). In traditional villages, tourists perceive the present moment, but also experience the village as it was thousands of years ago, in an immersive experience. The temporal and spatial immersion includes being captivated by beautiful scenery, becoming immersed in the atmosphere, transcending time and space, and enjoying the moment.

The manifestation of place spirit is intricately tied to the surrounding natural environment. The inherent allure of nature acts as the canvas for this connection. The physical surroundings of a village, encompassing the natural terrain, have a significant impact on the emotional bond between tourists and the location (Stedman, 2003a). The essence of place spirit can exemplify the harmonious rapport between humans and the natural world, enabling tourists to admire and articulate the splendor of nature. By visually perceiving the rural landscape, with villages nestled in mountains and by the water, tourists are captivated by the breathtaking scenery. In addition, when residents create a village, the atmosphere they cultivate reflects their nostalgia and return to nature. They experience the village deeply, immersing themselves in the spatial rural atmosphere and feeling the harmony, rustic charm, ecological authenticity, and warmth conveyed by the village ambiance. Through their interaction with the landscape and their experience of the place atmosphere, tourists enjoy positive emotional experiences.

There are numerous historical villages situated alongside the Nanxi River, which is a destination I thoroughly enjoy exploring during my leisure time (A11).

The Nanxi River region is renowned for its picturesque landscapes and idyllic charm, where the traditional way of life harmoniously coexists with the ancient village culture, and the customs of agriculture and education merge with the traditions of clans. The intimate connection between human existence and the natural surroundings offers boundless wisdom and aesthetic appeal (B11).

As tourists delve deeper into the experience, immersion is manifested by a loss of self-awareness and a distortion of the sense of time. In the present moment, the tourism space and atmosphere provided by traditional villages propel tourists to gradually lose their self-awareness, forget the passing of time and experience a pleasurable state in the present moment. Furthermore, as the emotional connection deepens, they transcend the barriers of time and space, resulting in an internal interpretation and resonance of emotions based on their identification with the village. It feels as if they are engaging in a silent dialogue with the ancient village, touching history and listening to stories from the past.

After a 1,000 years of wind and rain, today’s Cangpo still retains a strong Song Dynasty legacy. Strolling through the village, touching the nearly 1,000 years of history of the ancient village, every brick and tile records the stories of the past, as if traveling to Cangpo during the Song Dynasty (A4).

A 1,000 years of blandness breeds a deep cultural heritage, time slows down in Furong Ancient Village, and the ancient village stretches out the years like this, day after day, year after year (B18).

#### 5.2.2 Place conversion

Tourists visiting places of religious significance are referred to as spiritual travelers. From a phenomenological perspective, people seek meaning and life goals during their travels, expanding their religious experiences and making spiritual studies more diverse (Willson et al., 2013). Tourists seek the meaning attributed to villages and their personal values. Spirituality, mindfulness, faith, and physical devotion are experienced within the natural landscapes and social atmosphere.

Traditional villages are an ideal destination for modern tourists because they evoke their sense of direction, identity, and belonging through the spiritual ambiance of the village. The stress of modern life and work in a highly developed society can leave individuals feeling disoriented and disconnected. Tourism, thus, offers a secular pilgrimage that can fulfill the spiritual void. Villages possess a sense of sanctity, cultural legacy, and historical importance, which foster a sense of attachment to one’s homeland and ancestral land. Tourists may demonstrate a deep commitment in both their physical and emotional connection to the rural setting. The rural surroundings integrate the reverence and curiosity people have for nature, culture, and history, reflecting the harmony of the village and enabling tourists to physically relax and blend with the environment.

It is only after the baptism of time that the villages here are so deep, so wonderfully wonderful. “The oneness of heaven and humanity” is best interpreted here, and I have become a part of it (B18).

Tourists’ emotional expression is reflected in their spiritual beliefs and sense of belonging. Spiritual consciousness is widespread in the context of travel, but to what extent does rural tourism reveal spirituality? Villages create a unique atmosphere and convey a sense of place through tangible and intangible carriers, natural and social environments. When tourists visit, their minds are settled and shocked; their spirits find belonging and attachment, and their beliefs are strengthened and entrusted. As a result, the trip is endowed with a sacred meaning.

It is a quest for freedom, tranquility, leisure, and happiness in nature, and a search for a utopia of thoughts and spirits in ancient villages (B13).

Nostalgia is the entrance of the village. Nostalgia is the lush ginkgo trees at the entrance of the village, the Nanxi River flowing by the village, the winding paths sandwiched between the cobblestone walls, and the bulky wooden doors of the old mansions creaking. Traditional villages that carry historical and cultural memories also carry people’s nostalgia, leaving a spiritual pavilion for modern people to put their nostalgia in (A18).

Do not let life leave regrets. Do what you want to do. Do not let your feet stop. Chase your dreams (A9).

### 5.3 Behavioral willingness

#### 5.3.1 Tourist intention

The traditional villages of Nanxi River attract tourists from all over the country because of their uniqueness. When visitors come to explore the villages, they are captivated by the beautiful scenery and immersed in the history and culture of the villages, allowing them to relax and enjoy spiritual upliftment. As a result, tourists develop emotional attachment to the traditional villages and become loyal visitors. Conducting research on tourist behavior intentions is beneficial for enriching and improving tourists’ decision-making, and for providing recommendations for marketing practices for traditional villages.

Memory is a key factor for potential consumers when choosing a destination, and also influences tourists’ experiences of the destination and their sharing of the experience with others (Marschall, 2012). Tourists develop their own memories in traditional villages, creating impressions that can be shared with others. Potential consumers will consider the characteristics of the destination recommended by others when choosing where to go, and then test those characteristics out themselves. After experiencing a trip and developing a sense of fondness, loyal travelers are more likely to want to share their experiences. They will visit the villages again and recommend them to others, hoping that more people will appreciate and enjoy them.

The well-preserved ancient town, with its authentic charm, is highly enjoyable (C21).

The ancient village is small and exquisitely peaceful. Walking on the stone paths gives a sense of slowing down time. It is worth a visit in your leisure time! (C244).

Excursion in the rain, almost no tourists, mountain colors. I was very impressed by the beautiful colors of the mountains and the gentle rain. I will definitely come back to see it again when the weather is good (C62).

Yongjia villages have unlimited landscapes and scenery, (and are) a good place to visit. (I) would recommend it (C77).

#### 5.3.2 Conversion behavior

Place conversion is the belonging and attachment of the body and mind. It manifests in the behavior of tourists lingering and temporarily staying and exploring villages. Their experiences lead to them settling back into a new hometown. The concept of dwelling extends beyond mere shelter. People value the significance of their surroundings when it comes to dwelling. In the past, limited technology meant that people would choose to live in places that were safe and provided an abundance of food. Over time, more individuals have chosen to reside in cities. However, they have come to realize that houses made of concrete and steel do not provide them with a sense of identity and belonging. When visiting traditional villages, tourists have a profound experience of the local residents’ way of life. They form emotional connections to their spiritual beliefs and sense of belonging, expressed through admiration and envy of the locals, and through a sense of dwelling.

In ancient times, people made deliberate choices to settle in areas that were surrounded by mountains and water, resulting in the formation of villages. These settlements were established in natural environments, without the modern idea of constructing spaces. The spiritual essence of these places had a simple and natural allure. The inhabitants constructed houses using pebbles and trees, showcasing the close bond between humans and nature, and expressing their yearning for harmony with the natural world. Over generations, these villages developed their own unique histories and cultures, creating a distinct local atmosphere. When tourists visit these traditional villages, they feel an emotional connection with the place and experience its spiritual essence. The villages hold great significance for tourists as they admire and aspire to the lifestyle of the local residents. Hence, tourists often find themselves reluctant to leave these villages.

I truly envy the villagers here, who get to live a fairy-tale-like life in a picturesque setting (A2).

When I arrived here and stepped into that ancient village, filled with the intoxicating aroma of pure brew, with its ancient ponds, old trees, and old houses, I found it hard to leave and was completely captivated by its charm (B5).

Dwelling is a specific place where people belong, and better living is the ultimate expression of belonging. Urban industrialization means that space is extremely limited, and in crowded settings people’s spiritual pursuit of nature and freedom has moved from exploring Tao Yuanming’s imaginary land of joy and plenty to yearning for a poetic lifestyle. The Nanxi River aligns with this search for a spiritual home. Tourists’ sense of identity and belonging to the village deepens their desire to live there for a short period of time or to return to these hometowns for a longer period of time.

A quiet village by the Nanxi River, with a beautiful surrounding environment and picturesque scenery, a few days’ stay in a quiet place will make your mood better (C189).

The general feeling is that Yongjia is very scholarly, and there have been quite a number of famous scholars in its history, so if you have a couple of days to spare, it is very suitable for you to wander around and live in a small place (C31).

When I am old, how much I want to live in the village for a short time or return to my hometown for a long time. When I am old, I would like to leave a square acre of land in the ancient village of Nanxi River to grow vegetables, living an idyllic life of working at sunrise and resting at sunset. At night, sitting on the promenade of Lishui Ancient Street, chatting with my beloved, looking at the stars in the sky, swaying the bushel fan in my hand, growing old together (A12).

## 6 Conclusion and discussion

### 6.1 Conclusion

Based on the theory of place attachment, this study defined and explored the concept and essence of place attachment from the perspective of phenomenology of architecture. Qualitative research was used to develop a three-dimensional structural model of tourists’ place attachment that included situational perception, physical and mental conversion, and behavioral willingness.

(1) This study used the CAC theory to elucidate the mechanism of place conversion. Based on the CAC individual cognitive model, the structural mechanism of place conversion consists of situational perception, physical and mental conversion, and behavioral willingness, with temporal and interactive relationships between each part. Place conversion begins when tourists enter and perceive the rural space and atmosphere of traditional villages. The visitors are imbued with meaning through significance attribution and architectural art, and they develop a sense of identification with the village. Tourists’ emotions blend with the village. They become immersed in the space and time, and merge with the village environment. At the same time, tourists experience spiritual joy and attachment through their experiences. Finally, tourists develop a sense of loyalty to the place and a desire to live in traditional villages.

(2) The essence of the experience of place conversion is an exploration of immersion in time and space, and a sense of belonging in body and mind. This paper introduces the spiritual content of place in architectural phenomenology and clarifies the concept and composition of conversion of tourists in a place. Traditional villages are fascinating because of their harmony, simplicity, originality, and sense of familiarity. Traditional villages become a source of power for a place mainly because people can feel the presence of the spirit of the place. In ancient villages, the trees, bridges, streets, and archways are all quaint, especially the houses built with rough stones and logs. The courtyards are spacious, and the houses are open to the outside, allowing tourists to feel the lives of the residents through the streets and alleys, creating a warm atmosphere. The village blends seamlessly with the environment of the Nanxi River, creating a local atmosphere of a living village in its original state. Through immersion in time and space, tourists can communicate more deeply with the village. They perceive the spirit of the place, and find a sense of belonging in body and mind during this process. Tourists blend with the environment, are enveloped in nostalgia, seek the true meaning of life, and explore their own notions of utopia. The spirits of the people and the village blend and complement each other.

(3) Place conversion refers to the emotional connection people have with a particular location, which is often associated with a sense of belonging and identity. As technology advances and traditional villages gradually lose their charm, it can be challenging to find a sense of place and an authentic rural atmosphere. This phenomenon is known as the “loss of place” in the field of architecture. People often desire to escape the city and return to a more authentic way of life because of the lack of direction and identification in modern cities. Traditional villages offer tourists the opportunity to appreciate beautiful scenery, engage in conversations, exchange emotions, and experience historical and cultural aspects, perceived as meaningful. As a result, tourists develop a sense of identification and belonging, which makes them want to reside in these places.

### 6.2 Theoretical significance and implications

Research on the spirit of place mostly focuses on the field of architecture, with less research in the field of tourism. Further research on place attachment and loyalty is needed. Thus, the academic significance of this study is as follows: (1) The study introduced the phenomenology of architecture into the tourism field to demonstrate the interaction between spatial environments and individuals. Traditional villages, as existing spaces, combine the sense of direction and sense of identity of tourists. They convey a sense of place to tourists, and after emotional and spiritual processing, tourists embrace the villages. (2) Place attachment has been extensively studied, but place conversion – a new theory with higher dimensions – describes a spiritual sublimation based on emotional attachment to a place. Based on research on place attachment, this article further expanded the study of the spiritual experiential level from the perspective of architecture to understand the spiritual home of tourists. (3) Place conversion is a helpful concept for further research on tourist loyalty. Tourist loyalty emphasizes recommendations and revisits – linked to a love of the destination. The effect of place conversion can further extend to place residence, emphasizing tourist stays, living, and residing permanently, associated with spiritual attachment to rural destinations.

### 6.3 Practical significance and implications

This study aimed to construct the formation mechanism of place conversion through grounded theory, yielding practical insights for the preservation and development of traditional villages and the spiritual experience of tourists. Traditional villages embody the unique essence of China’s traditional agricultural civilization, encompassing natural landscapes and abundant historical and cultural information. They serve as living artifacts and museums of rural history and culture. The nostalgia they evoke represents the memories of the Chinese people toward the historical and cultural heritage of rural areas. Therefore, studying the spiritual core of a place and fostering a sense of place conversion is importance for safeguarding and passing on the original cultural, architectural, and historical aspects of traditional villages.

Research on place conversion is significant for finding rural memory points and returning to the essence of the countryside. In recent years, rural tourism has become an important part of the tourism industry. The history and culture of rural areas have gradually faded because of the vigorous promotion of urbanization in China’s modernization. The current development of modern rural areas in China creates challenges such as the uniformity of new rural architectural forms, the neglect of people’s social space with a focus on residential spaces, and the weak sense of identity and belonging among residents. Traditional villages are cultural spaces full of place spirit and collective memory – meaningful spaces that hold memories of “hometown.” The countryside is gradually becoming less like the countryside that Chinese people remember, and place conversion can awaken people’s place memory of Chinese-style countryside. Therefore, it is necessary to pass down and materialize the place spirit conveyed by traditional villages, pay more attention to the essence of place, and explore people’s sense of identity and belonging by creating meaningful places with local atmosphere. This study emphasizes the preservation and development of traditional villages as a way to return to the essence of the countryside and maintain the original authenticity of rural areas.

The study of place conversion has implications for the construction of the spiritual world of the tourist and the search for a poetic way of dwelling. Tourism differs from everyday life. It helps maintain a balance between the body and mind, reconstructing a beautiful spiritual home. The pressures and worries faced in everyday life are released during travel, allowing people to relax, enjoy, and find value in their lives. Therefore, exploring the relationship between traditional villages and individuals’ attachment and sense of belonging from the essence and connotation of tourism can further uncover the reasons why tourists choose to stay, live temporarily, or permanently, in a particular place. This has strong practical and guiding significance in maximizing the benefits and spiritual experiences of individuals through tourism – even transforming their lifestyle through tourism. Our study suggests that tourists should cultivate tourism as a necessary leisure activity and internalize it as a way of life.

### 6.4 Deficiencies and future research

The research on the formation mechanism of place conversion still needs further improvement. Firstly, this study is based on three traditional villages in Nanxi River, China, so it may not be applicable to other types of tourist destinations or countries, and its universality still needs to be tested. Secondly, this study involves multidisciplinary knowledge systems such as environmental psychology, sociology, architecture, and geography, and some aspects of the research may not be thorough enough. Thirdly, the study focuses on the formation mechanism of place conversion for tourists and does not consider the formation mechanism for residents, so the system needs further improvement. This study is an exploratory study based on the context of traditional Chinese villages, which constructs the formation mechanism of place conversion. In the future, we hope that the research results will be further tested and promoted. First, in terms of research methodology, in order to further develop the new concept of place conversion, quantitative confirmatory studies, such as the development of a scale to measure place conversion, can be conducted. Secondly, we hope to expand the research scope of place attachment to residents in future studies and explore the differences and connections between place conversion for residents and tourists. Finally, in terms of the study area, we hope that place conversion can be applied not only to study tourists’ spiritual and psychological experiences in traditional Chinese villages, but also to other tourist destinations that bring spirituality and soul belonging to people. For instance, Yunnan is one of the well-known tourism destinations in China. Its distinctive natural and social surroundings draw in a significant number of local and international tourists who come to visit, stay, and settle here. We aspire to delve deeper into the concept of place conversion in our future research and are eager to uncover new research insights. These issues need to be continuously improved in subsequent studies. We will continue to delve into and explore the research on place conversion in tourism in order to continuously expand its field and application scope.

## Data availability statement

The original contributions presented in the study are included in the article/supplementary material, further inquiries can be directed to the corresponding author.

## Author contributions

LX: Writing – original draft. LS: Writing – original draft. XW: Writing – review & editing. YW: Writing – review & editing. ZF: Writing – review & editing.
